# Stroke in Frail Older People

**DOI:** 10.3390/geriatrics2030024

**Published:** 2017-07-15

**Authors:** David G. Smithard

**Affiliations:** 1 Department of Electronic and Digital Arts, University of Kent, Canterbury CT2 7NZ, UK; david.smithard@nhs.net; Tel.: +44-1689-865856; 2 Clinical Gerontology, King’s College Hospital, London SE5 9RS, UK

**Keywords:** acute, stroke, old age, frailty, complications, ethics

## Abstract

The population is ageing, with the greatest proportional increase in those >80 years of age. Many of these people will be frail and at risk of stroke. Research has shown that the very old have much to benefit from hyperacute stroke intervention, but at the same time they suffer increased mortality. Their outcome following stroke and intervention is more often predicted by the presence of frailty rather than age alone. Intervention both in primary prevention and hyperacute stroke management needs to allow for preexisting morbidity and frailty in deciding what is and what is not appropriate, rather than an arbitrary decision on age. Frail older people are more likely to develop delirium and dysphagia combined with poor mouthcare and die, yet all of these issues are managed badly. An increased awareness of these complications of stroke in the frail older person is necessary.

## 1. Ageing and Stroke

**Figure 1:**
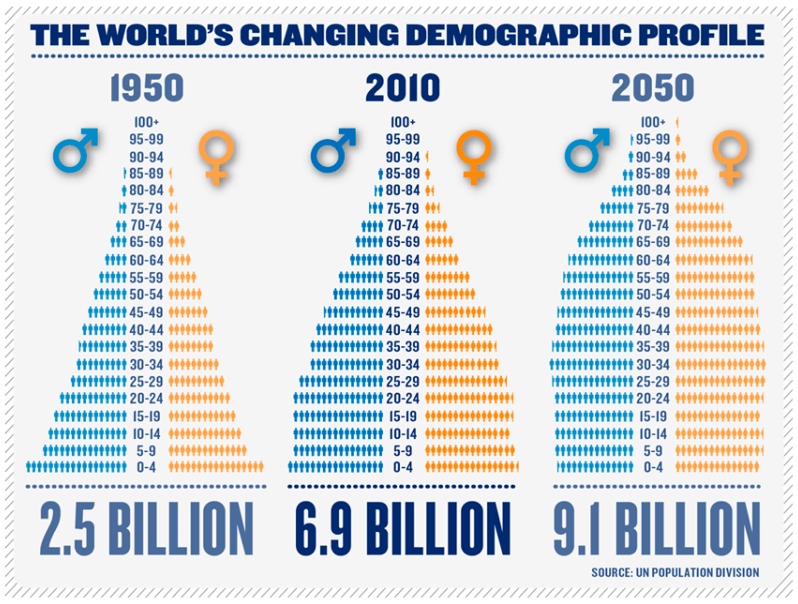
Changing age distribution, from the United Nations Population Division No 2012/4 December 2012. www.UNpopulation.org.

The world’s population is ageing, particularly in Western Europe and North America. By 2050 it is predicted that 35% of the European population will be over 65 years of age, albeit with an overall population decrease to 705 million from the present 728 [1,2]. Globally, those >60 years will number 2 billion by 2050, with those >80 years forming the fastest growing age group [3] (Figure 1) and 56.9 million will be >90 years of age [4]. As a consequence, the ratio of old to young will increase. Today, rather than age being celebrated, old age is feared and there is no desire to be old [5].

The definition of being old varies depending on the age of the respondent. It has typically been accepted that the age at which you retire will define you as old, but more and more people are retiring early and some people are working well into their ninth decade. Age can be defined biologically rather than chronologically. This is a more appropriate way forward and will help target treatments at appropriate older people.

The subset of the population that is increasing most rapidly is those who are deemed old or those over 80 years of age. Many of these people are frail (defined as a syndrome of decreased reserve and resistance to stressors resulting from a cumulative decline across many physiological systems leading to vulnerability and adverse outcome [6] with multiple co-morbidities, and high risk of fall, head injury and fractures [7,8]. Those who are severely frail have a six-fold increase in mortality [6,9].

This review will concentrate on the hyperacute management of stroke in frail older people, and some specific stroke complications (confusion, mouth care, swallowing and nutrition) that pertain particularly to this group of people. 

## 2. Stroke Epidemiology

In 2010 there were 16.9 million first-ever strokes, 5.9 million stroke-related deaths [10] and approximately 4 million stroke survivors [10,11] which may increase to 7.8 million by 2030 [12]. In 2010, Fonarow noted, from the Get With The Guidelines (GWTG) stroke database, that 168,000 stroke patients (33.5%) admitted to hospital were over the age of 80 years [13].

Of those admitted to hospital in the first quarter of 2016 (UK), the median age was 77 years, with those greater than 80 years comprising 39.7% of admissions (20,991) [14]; 11.7% of people had three or more comorbidities. This review will concentrate on the management of this frail older group of patients. 

## 3. Stroke Recognition

Stroke is easily recognizable when the symptoms are clear cut, for example, dense hemiparesis. Frail older people have multiple comorbidities [8] which may include previous stroke. 

The presence of multiple comorbidities may result in an atypical presentation of stroke, such that the diagnosis is missed [15]; milder symptoms may be ignored or brushed aside. Some ethnic groups retain a belief in stroke being caused by bad spirits and as such may go to local healers prior to, or instead of, attending hospital. All too frequently, older people are prescribed aspirin and not investigated further when a stroke with minimal signs or transient ischaemic attack has occurred.

As a consequence, potential treatments (thrombolysis, thrombectomy) may denied, or the presence of decompensation may result in a “mimic” presentation; there is a subsequent danger of an intervention that is not required.

The default position needs to be: investigate for stroke and treat unless there are very good reasons not to.

## 4. Pre-Stroke Function

Although getting old is not a disease process [16,17], very old people frequently have multiple comorbidities/long-term conditions [18,19] that are complex, with potentially coexistent medical, functional, psychological, and social needs [9,20] which may compromise their ability to live independently prior to admission with stroke. 

Many older people will not have a “normal” brain [21]; for many, a routine Computed Tomograph (CT) brain scan either before or after stroke reveals diffuse periventricular ischaemia [22]. These are associated with dementia, infection, Parkinsonism, motor disorders and stroke recurrence.

## 5. Stroke Aetiology and Risk Factors

With increasing age and frailty, there is likely to be a logical and apparent aetiological agent for the stroke and rare causes are less likely to be a concern. Age is a major risk factor for stroke, it is unmodifiable and will come to all, older people are exposed to the same risk factors as younger adults.

Stroke may be classified by many different systems, ischaemic vs. haemorrhage; clinical symptoms [23] or by aetiology [24]. The majority of strokes, irrespective of age, are ischaemic in origin, and older people are more likely to suffer from cardioembolic rather than atherothrombotic strokes and large artery (total/partial anterior) rather than small vessel (lacunar) strokes [6]. Rarer forms of stroke tend not to present in old age, though stroke mimics (migraine, psychological) should still be considered.

The medical management of risk factors in the frail older person is complicated by polypharmacy and the evidence of benefit over risk of adverse effects. Some are unable to tolerate the anticholinergic burden [25,26] associated with many medications. Consequently, treatment guidelines should be noted and amended on an individual basis, depending on pre-existing medication, functional ability and life expectancy. Hypertension, atrial fibrillation, raised cholesterol and the use of antiplatelet agents are discussed briefly below as these can be areas of contention in the management of frail older people.

## 6. Hypertension

Hypertension remains a major risk factor for stroke [6]; the studies confirm that treating older people for high blood pressure can reduce the risk of stroke [27]. However, experience suggests that not all frail people are able to tolerate antihyperetensive medication, due to increased risk of falls, postural hypotension and light headedness/ confusion, and renal function issues [28]. Won et al. [29] in their review comment that the Hypertension in the Very Elderly Trial (HYVET) study is the only one to document any benefit in significantly lowering the blood pressure in frail older people.

## 7. Atrial Fibrillation

Atrial fibrillation occurs more commonly in older people, secondary to ischaemic heart disease (in the Western World). Older people are in the high risk group according to the CHADVASC scores [30] predominantly on the basis of their age. The risk of stroke is up to 10% in those >80 years. Perera et al. [31] found that frail patients were more likely to have embolic stroke if not receiving anticoagulant therapy (Relative Risk (RR) 3.5 95%, Confidence Interval (CI) 1.0–12.0), with a non-significant increase of major haemorrhage (RR 1.5 95% CI 0.7–3.0) and a greater risk of mortality (RR 2.8 95% CI 1.2–6.5). 

A risk of falls is a relative contraindication with respect to anticoagulation, but many non-specialists think otherwise. Poor concordance with medication is a greater risk (intermittent taking of medication will increase risk of bleeding with no benefit from stroke prevention). 

Where possible and safe to do so, they should be offered treatment with warfarin or a New Oral Anticoagulant (NOAC) (e.g., Rivoraxaban). NOACs are as effective as warfarin and may reduce the risk of intracranial haemorrhage. They have fewer interactions and therefore may be the better choice, particularly if not severely frail [32]. Concerns have been raised regarding the lack of monitoring and whether, despite fixed dosing, some form of monitoring may be required. In some undernourished patients, a NOAC may be a wiser choice as absorption is likely to be affected by diet [30].

## 8. Antiplatelet Use

Antiplatelet agents are commonly used for the primary prevention of cardiovascular disease and the secondary prevention of cerebrovascular and cardiovascular disease. There are several antiplatelet agents that can be used as well as aspirin. Clopidogrel is increasingly used and may be preferred where other vascular fields are affected. Trifusal and cilostazol may be used but are not universal. Dipyridamole as a controlled release agent has been recommended, (but is not contained in all up to date guidelines) and due to its hypotensive effects may increase the risk of falls in this age group. 

In frail older people, there is no contraindication, but in the case of aspirin a proton pump inhibitor should be co prescribed to reduce the risk of gastric side effects. There is also a possibility that the stroke protective effect of the antiplatelet may be reduced due to reduced levels of plasma aspirin esterase [33], and conversely, there is an increased risk of haemorrhage if there are significant levels of periventricular lucencies and hyperintensities (minor bleeds) present on the CT scan [34].

## 9. Cholesterol

Although a raised cholesterol or cholesterol ratio is a risk factor for stroke disease, the benefits of aggressive management in very old age/frailty are uncertain [35]. The National Institute of Health and Care Excellence do not publish evidence for those over the age of 85 years. 

Frailty and age (life expectancy) will be more likely to have an effect on prognosis than any elevation of cholesterol. Therefore, the use of medication for primary or secondary prevention will need to be questioned and trial evidence treated with circumspect, especially if results have to be extrapolated from data obtained in studies where frail people have frequently been excluded [29]. 

## 10. Access to Stroke Care

There is a concern that with increasing age, older people will be denied access to stroke services [13,36] when a referral would have been appropriate [37,38,39]. There is frequently a combination of factors such as the patient not seeking or delaying seeking help; health professionals not referring the patient to hospital and hospital-based staff not treating or recognising stroke in older people due to the presence of dementia or other neurodegenerative disease. Older people may be offered a separate rehabilitation pathway that appears on the face of it, less aggressive.

Even when referred, there has been a suggestion that those over 80 years are less likely to access intensive care [40] and in 1999, the Biomedical (BIOMED) Stroke study [36] study reported that older people were less likely to be investigated. If they were living in institutions, there was an independent increase in disability (Odds Ratio (OR) 2.11 CI 1.22–4.45) and handicap (OR 7.04 CI 1.62–30.7).

### Hyper Acute Stroke Management

The hyperacute management of stroke has been proven to reduce the disability associated with stroke [39,41,42]. Recent data has shown that the benefits seen in the “younger” population is the same or greater in older people (>80 years of age). 

**Figure 2:**
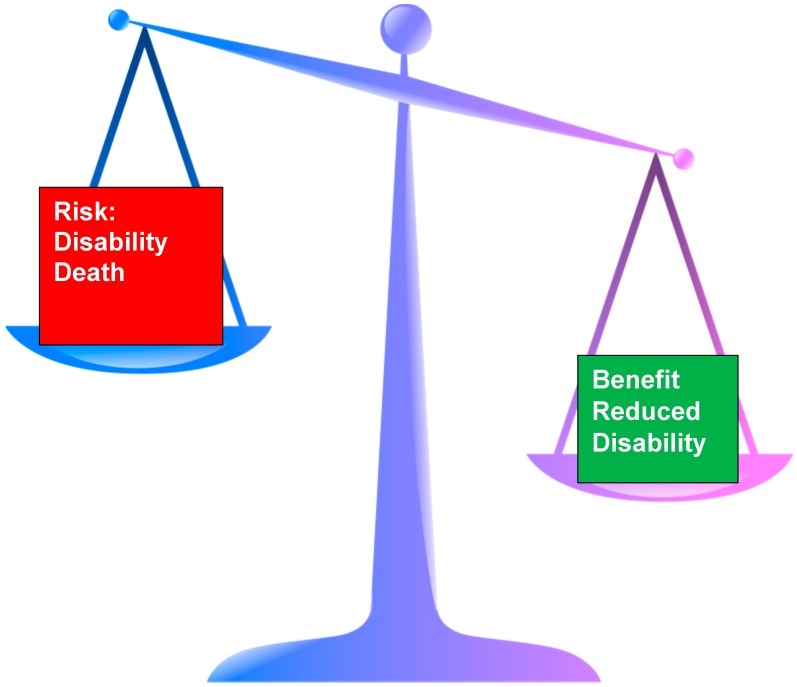
Schematic representation of risk vs. benefit in hyperacute stroke treatment in frail older adults.

Frailty is a better predictor of outcome from stroke than age alone [7]; as a consequence, the approach to the hyperacute management of stroke will need to be tempered depending on the degree of frailty present prior to the stroke (Figure 2). 

The following discussion covers the more common interventions in the management of hyperacute stroke. 

## 11. Intravenous Thrombolysis

The early trials on thrombolysis [43], did not recruit significant numbers of very old subjects; more recent work has supported the relative safety of thrombolysis in a selected group of older people [44,45]. The number of people that need to be treated for benefit is 8 compared to 36 in younger patients. However, in people >85 years of age, despite the increase in potential benefit, mortality is double that of those <59 years [46]. The Safe Implementation of Thrombolysis in Stroke - International Stroke Thrombolysis Register database, reported that the increased mortality in older and frail people was more likely to be due to coexisting morbidity than thrombolysis per se [47].

## 12. Endovascular Thrombectomy

Endovascular thrombectomy has entered mainstream practice in the acute management of stroke, though in the UK it is not, at this moment in time, universally available. It is feasible to undertake this intervention in very old people and to achieve comparable reperfusion rates, however 90-day outcome (death and disability) is worse, both in functionality and an increased mortality [48] (10% vs. 40%) [49]. The poor outcome appears to be associated with pre-stroke function and stroke severity (as indicated on the National Institue of Health Stroke Score (NIHSS) and Alberta Programme Early CT (ASPECT) scores) [50,51]. Distal aspiration of the clot may infer no additional benefit [49] Hassan noted that despite an equal revascularization rate, the utilization of endovascular removal of the clot was used less frequently in those over the age of 80 years. This may have been in part due to careful selection of very old and frail stroke patients [52]. The Total Health Risks in Vascular Events (THRIVE) score is a useful tool to predict outcome from endovascular interventions [53]. 

## 13. Clot Evacuation

Intracranial haemorrhage may be due to vessel rupture secondary to an aneurysm, amyloid angiopathy, or arteriovenous malformation. In many cases the treatment will be expectant. Where there is a significant intracerebral haematoma, surgical intervention may be considered.

Evacuation of a clot following intracerebral haemorrhage does not show any benefit unless there are signs of increased intracranial pressure [53,54], therefore clinical condition rather than age will be the arbiter. The use on minimally invasive surgery with thrombolysis [55] has shown some benefit, but all participants were below the age of 75 years. 

## 14. Hemicrainectomy

More radical approaches to stroke treatment include hemicrainectomy; original studies focused in the age group <60 years of age [56,57]. Even though benefit was shown, people were often left severely disabled. Recent studies such as DESTINY II [58] have shown that older people will benefit. The DESTINY II trial of decompressive hemicraniectomy for older patients with severe space-occupying middle cerebral artery territory infarction has shown a substantial survival benefit for patients over the age of 60 years [58] akin to that seen in young patients [59].The 30-day mortality rate was significantly higher in the group that was >70 years of age (0% vs. 60%; *p* = 0.01) than in the group that was 61–70 years of age [60]. Studies involving significant numbers of patients >80 years have not been undertaken and therefore conclusions cannot be drawn other than to suggest caution and the need to enrol people into research trials where possible. 

Stroke guidelines both in the USA [40] and UK [41] recommend offering hemicraniectomy to appropriate older people being aware of the potential for severe disability but patients should not be excluded from treatment by age alone. 

## 15. Carotid Endarterectomy/Carotid Stenting

Similarly, with carotid endarterectomy [61], making a decision based on biology and not chronology will help determine who should and should not be offered this treatment. Researchers have noted [62] that although the risks were greater in those >80 years of age, the outcomes, in those undergoing surgery, remained comparable to those of younger patients. Guidelines would suggest, at this time, surgery should be the intervention of choice.

## 16. Outcome

Longer-term survival may be more linked with age, as life expectancy is less. For instance, those born in the 1920s–1930s may have only been expected at birth to live into their late 70s, so survival into their 80s is remarkable on its own. Increasing age is an accepted risk factor for ischaemic stroke, but there is a debate as to what affect age has on recovery. There are few studies that systematically look at this, and those that do, have provided conflicting results. However, pre-stroke functional ability and cognition are associated with outcome [15,20].

The better the pre-stroke cognitive function at the time of stroke, the better functional outcome [54]. Stroke recovery requires neuroplasticity to occur, however animal studies have suggested that with increasing age there is a reduction in neurogenesis in basal hippocampus regions compared to striatal-like regions [63]. The presence of an ischaemic brain [22] pre-stroke status will affect post-stroke recovery; particularly executive functioning and memory task processing will be affected [22]. The presence of a high load of periventricular ischaemia would suggest a reduction in brain reserve, and hence the ability to rewire the brain [64,65] may be reduced [66]. 

**Figure 3:**
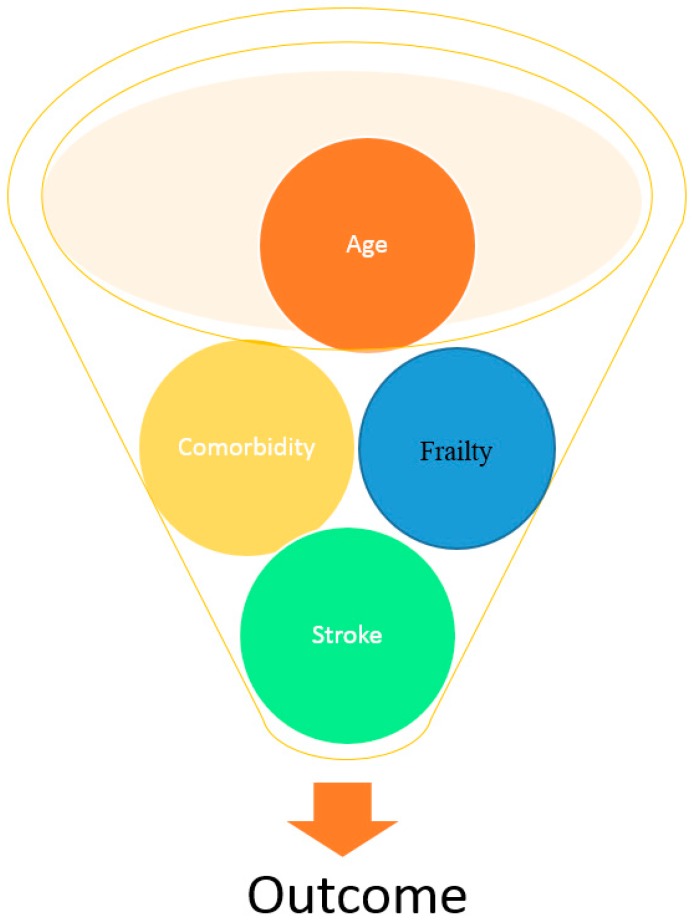
The interplay between age, frailty, comorbidity and stroke on outcome.

The potential for a good outcome following thrombolysis/endovascular intervention/hemicrainectomy is greater in older people than young. However, despite the positive outcomes, all studies report that with increasing age, and comorbidities (e.g., as demonstrated using the THRIVE score) [67], there is an increased risk of poor functional outcome and mortality [41] (Figure 3).

Randomised studies and case series often recruit a fit older population (for instance the third International Stroke Trial (IST 3) recruited only those people >80 years with limited comorbidities) [68]. This results in the question as to whether the results are generalizable and applicable to frail people and underlines the importance of careful selection. 

Although some of the outcome is related to pre-stroke function/disability, this raises the question as to whether age is actually a predictor or a surrogate marker for the presence of comorbidities, less aggressive rehabilitation, lower expectations and earlier discharge due to lower goals.

With these acute treatments available, there is a danger that with the wrong selection, more older people will survive but be more dependent. This may result in a significant proportion of very old people being discharged to nursing/residential home care rather than going home [15,65].

Ergeletzis [69] noted that older people had a shorter length of stay in hospital (*p* < 0.05) and also had a lower functional recovery despite completing the rehabilitation programme. For every 10-year increase in life after 60 years of age there is a 7% reduction in gain on the Barthel Index following rehabilitation. Those <70 years of age are more likely to be able to walk [70]. Those over 80 years gained less functionally able from a mobility perspective [39,63], Kong et al. however, report that despite age, significant gains/improvements can be expected, though expectations of the end result/total recovery may need to be lower [71]. In terms of longer-term outcome, five years after stroke very old age predicted mortality and nursing home placement [15,40].

## 17. Ethical Issues

Management of stroke and its complications are fraught with ethical and moral questions at the best of times. One of the greatest challenges is to provide the appropriate treatment to someone irrespective of age. To provide equity of access, and although this has improved over the years, disparities remain [13].

In frail older people, this is more so. Some frail older people may not wish to undergo aggressive investigation and intervention; where previous wishes have been expressed/documented, these need to be accounted for and respected.

However, the presence of confounding comorbidities, pre-existing physical and cognitive functioning play a major role on predicting outcome from acute stroke. 

Just because there is a treatment available it does not mean that that treatment needs to be or should be offered to everyone. 

The decisions to start, stop or not initiate treatment need an individualized approach. The use of enteral feeding, antibiotics, and parenteral fluids are all important bridges that may have to be approached and crossed.

## 18. Life Events

A stroke is a major life event. Recovery is often fashioned by the experience of others and the adaptability of the person to their life path. As people get older, stroke may be seen as something to be expected, a common and natural occurrence. For others it is a devastating consequence and without support from a neuropsychologist or psychiatrist they may struggle to adapt and to recover [72].

One concern is the acceptance of the sickness role, and that the family may be over-supportive and inhibit independence out of love and concern. There are times when this will be oppressive and have a negative influence, resulting in someone who becomes more dependent on others than they should be.

Quality of life after stroke is affected/influenced by many factors; adaptation to the new situation is a major player. With the emphasis on home being the best discharge destination, it is interesting to note that Brajkovic et al. [73] found that those resident in nursing homes reported a better quality of life. Each person has networks; family, friends, church and so on. As one ages these networks get smaller [74].

## 19. Death

Stroke is the third leading cause of death in the West. The one-month mortality following stroke is as high as 20% [41,42] and in some health services this may be higher depending on care standards and case mix. It is not ethical to provide treatment where the benefit is minimal and this is outweighed by the potential harm. This includes the continuation of treatment or commencement where it is futile.

With increasing age, death becomes more prevalent. Living to old age is in itself a measure of successful ageing. As age increases, older people think more about dying, not the actual fact of dying, but the process of dying rather than death itself [75].

Supporting people who are dying will require decisions around nutrition/feeding, hydration pain relief and the management of secretions.

## 20. When to Consider End of Life Care

It must be remembered that everyone has a right to life; but they have a right to a good death and a right not to be tortured [76]. Providing interventions that are not indicated or are of no benefit can be construed as common assault (particularly if the patient had expressed a desire not to have such intervention) or at worst, torture, and is unethical just as much the denial of treatment may be unethical.

Treatment decisions are medical but need to be made in conjunction with the other interested parties, in particular the patient, if they have the capacity to do so, or their legal appointed representative. Otherwise other family members can only inform the decision-making process. All members of the multidisciplinary team must be involved to come to a decision regarding the best interest of the patient.

## 21. Stroke Complications

Stroke is itself a chronic condition and is associated with many frequently unmet needs including isolation, depression, poor quality of life, fatigue and apathy [12,77,78] With time and increasing age, physical functioning may deteriorate, but social and emotional domains may improve [79]; aphasia appears to have the largest negative effect on outcome [80].

Complications following stroke are common and include visual disturbance, dysphagia, chest infection, venous thromboembolism, confusion, and bowel and bladder disturbance. The management of all complications is important but beyond the scope of this paper. A few, more specific complications that are prevalent in frail older people and may be difficult to manage are discussed below.

## 22. Confusion

Acute confusion or delirium is common in frail older people. Stroke does not present with confusion, but severe dysphasia/dyspraxia of speech may be mistaken for a confusional state. 

Recovery from delirium may take many months or may not occur even without the presence of stroke. 

The presence of delirium may be should trigger a search for another cause such as infection [81], constipation, metabolic abnormalities, hypoxia or medication [82]. Without treatment, delirium carries a significant mortality over and above that for stroke. Stroke patients with delirium have higher inpatient mortality (OR.; 4.71; 95% CI.; 1.85–11.96) and mortality at 12 months (OR.; 4.91; 95% CI.; 3.18–7.6) compared to those without. Patients with delirium also tended to stay longer in hospital compared to those who did not (mean difference, 9.39 days; 95% CI.; 6.67–12.11) and were more likely to be discharged to a nursing home or other institutions (OR.; 3.39; 95% CI.; 2.21–5.21) [82].

## 23. Mouth Care/Swallowing/Nutrition

Mouth care is frequently neglected on stroke units and general wards (particularly where enteral nutrition is being administered). At present there is an international effort to raise the awareness of the need for good mouth care. Frail older people, when ill, after stroke may not be able to keep their mouths clean [83,84]. Poor mouth care results in an increased risk of pneumonia from aspiration. The best way of managing this is good mouth care [84,85]. The use of antibiotics does not help [86,87], though metoclopramide may be of some benefit [88]. 

Dysphagia/swallowing problems affect many people following their stroke, and there is a likelihood of an increased prevalence in older age due to co-existing neurological problems or presbyphagia [89,90,91,92]. Other chronic conditions such as cardiac or lung disease can lead to dysphagia, due to respiratory problems.

As people age, the mechanics of swallowing will change [93,94]. Many older people have noticed that they have reduced the amount they eat, or the texture, due to fatigue or coughing.

Dentition and false teeth can really impact on the ability of older people to eat food, compounded by the dislike of the food served. Many older people are missing teeth, have dentures that do not fit, or have gum disease

Nutrition is important. Many frail older people are living on the edge of undernutrition, 24.6% of community frail older adults are malnourished and a further 62.4% are at risk [95,96]. These figures may be worse within nursing homes; up to 80% may have feeding problems [95,97,98]. Following stroke, nutritional status deteriorates and may take many months to recover to baseline [99]. If baseline is already low, then these frail older people will potentially do badly and will be prone to infection and pressure area breakdown.

Pre stroke, many older people will have been suffering from either overt dysphagia or presbyphagia [94], with the incidence increasing in the presence of dementia and many other long-term conditions [94]. There may have been a need for modification of diet either consciously or subconsciously. This may be exacerbated following the stroke. Apart from reduced ability to follow or complete rehabilitation exercises [100], (that alter swallowing physiology) many will not enjoy modified fluids or solids. There is a risk that people will become dehydrated [101,102,103] and at present there is a lack of standardisation of the administration of thickeners [104,105].

## 24. Conclusions

Age is not an absolute contraindication for acute and hyper acute intervention following a stroke. Frailty and multiple co-morbidities are more likely to influence outcome than the intervention itself. The management of hyper acute stroke in frail individuals is fraught with ethical dilemmas, especially surrounding the appropriateness of treatment in the context of poor outcome and likely death. Therefore the approach to the management of stroke with frail people should take a bespoke approach. Confusion, dysphagia, poor mouth care and poor nutrition are common complications following stroke particularly in the presence of frailty. Vigilance and appropriate early intervention is recommended.
